# Circulating microRNAs and Kallikreins before and after Radical Prostatectomy: Are They Really Prostate Cancer Markers?

**DOI:** 10.1155/2013/241780

**Published:** 2013-10-09

**Authors:** Maria Giulia Egidi, Giovanni Cochetti, Maria Rita Serva, Gabriella Guelfi, Danilo Zampini, Luca Mechelli, Ettore Mearini

**Affiliations:** ^1^Department of Medical-Surgical Sciences and Public Health, Faculty of Medicine and Surgery, University of Perugia, Loc. S. Andrea delle Fratte, 06156 Perugia, Italy; ^2^Department of Biopathological Science and Hygiene of Food and Animal Production, Faculty of Veterinary Medicine, University of Perugia, Via San Costanzo 4, 06126 Perugia, Italy

## Abstract

The aim of our study was to monitor serum levels of two miRNAs (miR-21 and miR-141) and three KLKs (hK3/PSA, hK11, and hK13) before and 1, 5, and 30 days after radical prostatectomy, in order to characterize their fluctuations after surgery. 38 patients with prostate cancer were included. miR-21 and miR-141 were quantified through real-time PCR, while ELISA assays were used to quantify hK3 (PSA), hK11, and hK13. Both miR-21 and miR-141 showed a significant increase at the 5th postoperative day, after which a gradual return to the preoperative levels was recorded. These findings suggest that miR-21 and miR-141 could be involved in postsurgical inflammatory processes and that radical prostatectomy does not seem to alter their circulating levels. Postoperative serum kallikreins showed a significant decrease, highlighting the potential usefulness of kallikreins apart from PSA as potential prostate cancer markers.

## 1. Introduction

Prostate cancer (PCa) is the most common male tumor in Europe and USA. The introduction of prostate-specific antigen (PSA, i.e., hK3) in the diagnostic management has allowed an earlier detection and thus the possibility to treat more cancers at a precocious and localized stage, improving the cure rates. Kallikreins are a family of peptidases widely expressed in various tissues, and they rule various physiological events, such as blood pressure homeostasis, skin homeostasis, and semen liquefaction. The proposal of kallikreins besides PSA as cancer biomarkers has been already reported, on the basis of the alterations in expression of many members of this family in several cancers [1–3].

microRNAs (miRNAs) are small noncoding single-stranded RNAs controlling the expression of protein-coding transcripts: each miRNA targets several genes at the posttranscriptional level [4]. About 10–30% of all human genes are regulated by miRNAs [5]. In the last decade, microRNAs have been studied as potential markers for almost every type of cancer [6]: miRNAs locate at cancer-related genomic regions or in fragile sites, and this feature suggests their potential role in tumorigenesis [7]. In 2007, Porkka performed the first profiling of miRNA involved in prostate cancer [8]. miRNAs and KLKs are characterized by a high stability in the serum and for this reason they are both ideal candidates for noninvasive assays.

The first end point of this study was to compare preoperative expression levels of three kallikreins (hK3/PSA, hK11, and hK13) and 2 miRNAs (miR-21, miR-141) with postoperative ones, in order to evaluate if a specific correlation between prostate cancer and these markers exists. The second aim was to evaluate their diagnostic power comparing the preoperative values in prostate cancer patients with healthy ones.

## 2. Materials and Methods

### 2.1. Experimental Design

**Figure 1:**
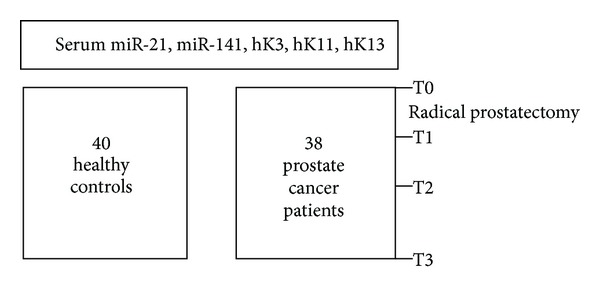
Overview of the experimental design. T0: presurgical phase; T1: 1 day after surgery; T2: 5 days after surgery; T3: 30 days after surgery; C: control.

This study was approved by the Institutional Internal Review Board of Santa Maria Hospital, Terni. 38 patients who undergone radical prostatectomy for localized prostate cancer were enrolled consecutively from September 2011 to April 2012. 40 subjects were selected as healthy controls: their mean age was 39 years, mean PSA was 0.56 ng/mL, and they have no history of prostatitis or other urologic pathologies. The demographic characteristics of all prostate cancer patients are reported in Table 1, and the experimental design is shown in Figure 1. Expression levels of miR-21 and miR-141 were measured through real-time PCR, while ELISA assays were conducted on the same serum samples to quantify hK3 (PSA), hK11, and hK13. In prostate cancer patients, serum levels of miRNAs (miR-21 and miR-141) and kallikreins (hK3, hK11, and hK13) were measured at four time points: 1 day before surgery (T0) and 1st (T1), 5th (T2), and 30th (T3) postoperative days (Figure 1).

### 2.2. Serum Collection and Storage

 Serum samples were all obtained from subjects subscribing informed consent. All patients underwent fasting blood withdrawal at one day before surgery (T0) without receiving any drug treatment. Blood was withdrawn into Vacuette Z Serum Sep Clot Activator (Greiner Bio-One). Collection tubes were gently inverted five times to ensure full contact with inner procoagulant surface, and coagulation process was allowed maintaining vacuette for 30 minutes at RT. After centrifugation (2000 ×g, 10 min), aliquots were immediately stored at −80°C until use.

### 2.3. RNA Isolation

200 *μ*L of serum was used for the extraction of total RNA with Total Purification Kit (Norgen Biotek Corp., Ontario, Canada), following the instructions for plasma and serum, with minor modifications. Total RNA was quantified by Qubit RNA assay (Life technologies) and stored at −20°C until use. 

### 2.4. Reverse Transcription

4 *μ*L of purified RNA (20 ng) was reverse-transcribed using the miRCURY LNA Universal RT miR PCR, polyadenylation, and cDNA synthesis kit (Exiqon). RNA spike-in control (UniSp6 RNA template, provided with the cDNA synthesis kit) was introduced in RT mix immediately before retrotranscription in a 20 *μ*L cDNA reaction and served to monitor cDNA synthesis and PCR efficiency.

### 2.5. Real-Time PCR 

Primers were designed based on mature miRNA sequence (miRCURY LNA specific PCR primer set, Exiqon system). Each sample was run in triplicate, and the results were averaged; no-template controls were included in the analysis. Exiqon miRCURY LNA Universal RT microRNA PCR SYBR Green master mix has been used to amplify cDNAs. All PCR reactions were performed on a Bio-Rad iCycler Real-Time PCR system. Three miRNAs (miR-93, -103, -191) were tested for normalization according to the manufacturer's recommendations for serum/plasma applications [9]. 

### 2.6. ELISA Assay of hK11 and hK13

250 *μ*L of serum was used for measurement of hK11 and hK13 with sandwich ELISA (human KLK-11 and KLK-13 pair sets ELISA kits, Sino Biological Inc., Beijing, China). ELISA microplates were separately coated with 100 *μ*L of diluted of affinity-purified rabbit mAbs anti-hK11 or anti-hK13 (2 *μ*g/mL in 5 mM carbonate-bicarbonate), incubated overnight at 4°C, and washed three times with TBS containing Tween 20 0.05% (300 *μ*L/well). Solid phase blocking was performed adding TBST containing BSA 2% (300 *μ*L/well) and incubating 60 min at RT. Wells were then washed three times, and 100 *μ*L/well of serum samples was incubated for 60 min at RT. Plates were washed again as above, and 100 *μ*L/well of anti-rabbit IgG antibody HRP labeled (1 *μ*g/mL) was dispensed and incubated for 60 min at RT. Plates were once more washed, and 100 *μ*L/well of TMB solution (Sigma) was added and incubated for 20 min, before stopping with 100 *μ*L/well of H_2_SO_4_ 1 N and measuring OD at 450 nm on a microplate reader (Infinite 200, Tecan, Männedorf, Switzerland). hK11 and hK13 serum levels were referred to a standard curve of recombinant human kallikreins 11 and 13. 

### 2.7. Total PSA Assay

Total serum PSA measurements were performed through Advia Centaur automated system (Siemens Health Care Diagnostics, Victoria, Australia). The two-site sandwich immunoassay consists of polyclonal goat anti-PSA antibody and a monoclonal mouse anti-PSA antibody.

### 2.8. Statistical Analysis

Tukey's Multiple Comparison Test was used to compare the serum samples obtained before and after surgery and to compare the difference in the serum miRNA and hKs expression between the cancer group and the healthy control group. The significance was set as *P* ≤ 0.05.

Receiver Operating Characteristic (ROC) curve analysis was performed to estimate the diagnostic accuracy of miR-21, miR-141, hK11, and hK13. Logistic regression analysis was performed to evaluate the diagnostic accuracy of the combination of miRNAs.

## 3. Results

### 3.1. Serum hK3, hK11, and hK13 before and after Radical Prostatectomy

**Figure 2:**
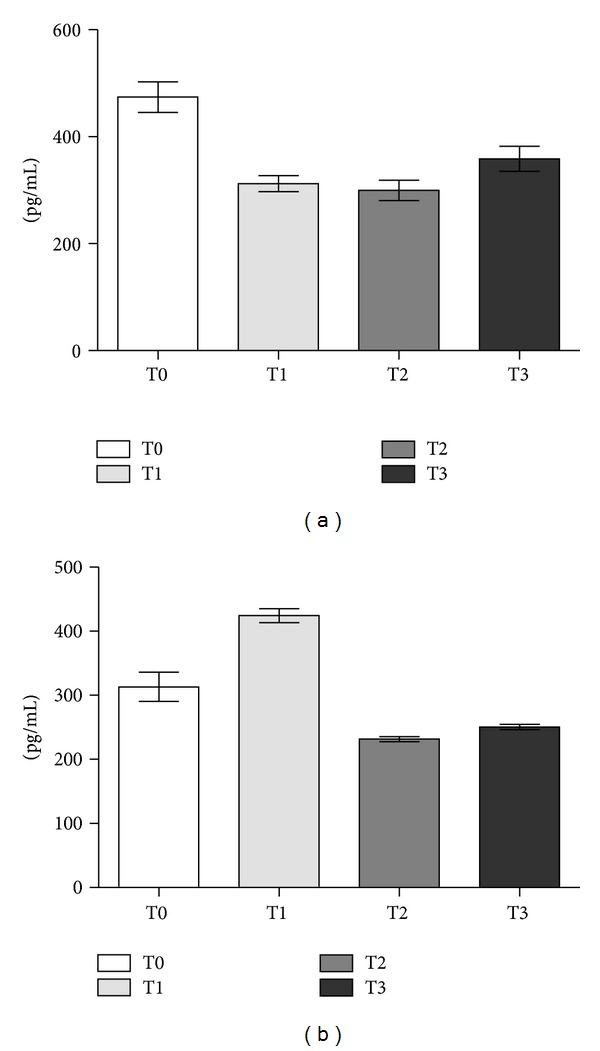
hK11 (Panel a) and hK13 (Panel b) levels (pg/mL ± SE) by ELISA in sera from patients with localized prostate cancer. T0: preoperative time; T1: 1st postoperative day; T2: 5th postoperative day; T3: 30th postoperative day.

**Figure 3:**
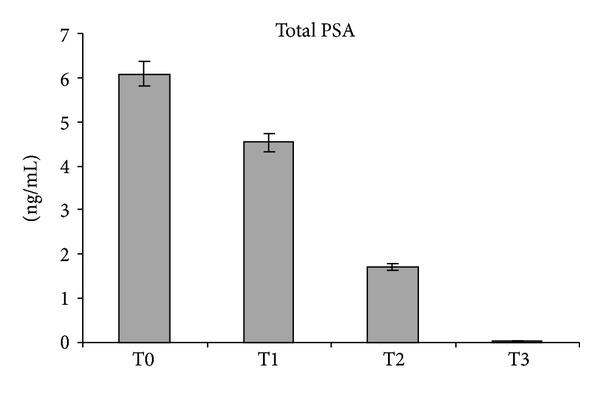
PSA levels expressed as ng/mL ± SE. T0: preoperative time; T1: 1st postoperative day; T2: 5th postoperative day; T3: 30th postoperative day.

hK11 levels significantly decreased after surgery (T0 > T1, *P* < 0.05) and remained substantially unmodified at T2 and T3 (*P* > 0.05) (Table 2). The marked decreased of hK11 at the first postoperative day resembled that of PSA (Figure 2). Contrary to PSA which progressively decreased to finally fall down to zero at T3 (Figure 3), hK11 showed a slight increase at T3, although this was not statistically significant (*P* > 0.05). 

In contrast, serum hK13 levels significantly increased at T1 (T0 < T1, *P* < 0.05) and finally stabilized at lower levels with respect to preoperative values (T2 < T0, *P* < 0.05) (Figure 2). As seen for hK11, hk13 did not decrease further at T3 (T2 versus T3, *P* > 0.05).

#### 3.1.1. Comparison of Serum hK11 and hK13 between Prostate Cancer Patients before Surgery (T0) and Healthy Controls (C)

**Figure 4:**
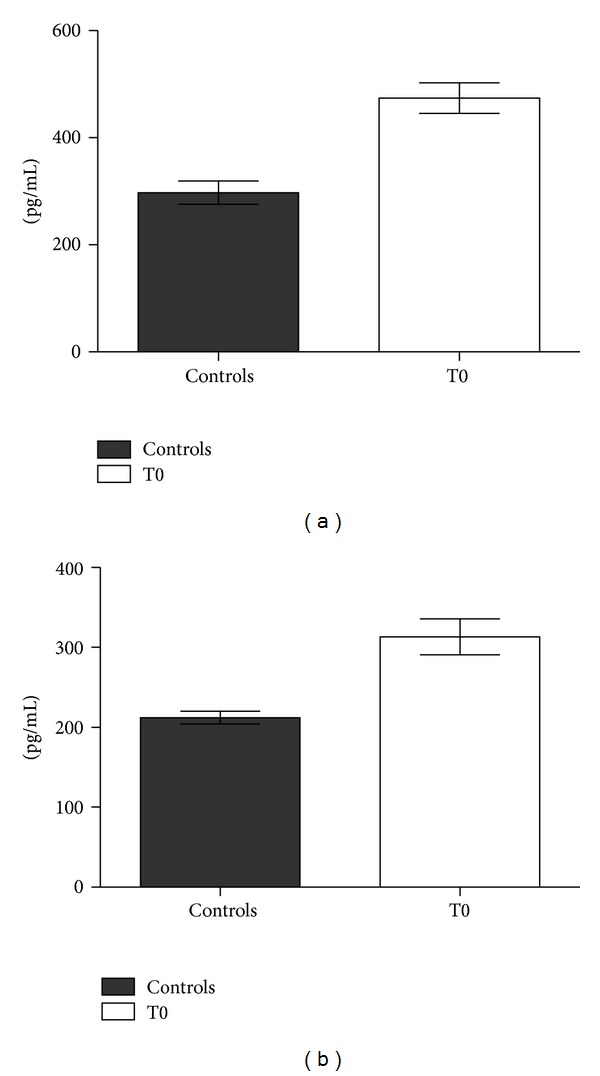
hK11 (Panel a) and hK13 (Panel b) levels (pg/mL ± SE) by ELISA in sera from patients with localized prostate cancer. C: control; T0: preoperative time.

**Figure 5:**
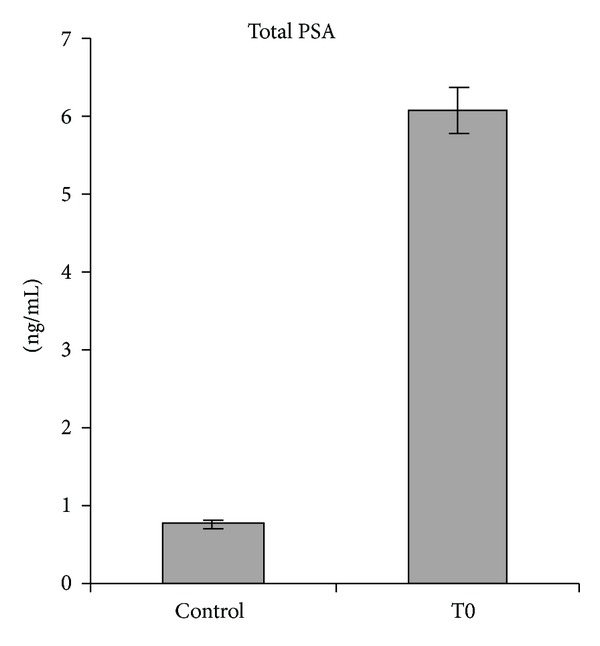
PSA levels expressed as ng/mL ± SE. C: control; T0: preoperative time.

Serum levels of hK11 and hK13 were significantly higher (*P* < 0.05) in serum from prostate cancer patients at T0 with respect to healthy controls (Figure 4 and Table 3), similarly to PSA (Figure 5).

Serum levels of hK11 and hK13 in control group were also compared with values obtained from prostate cancer group at postoperative sampling times (T1, T2, and T3, see Table 4). Only serum levels of hK13 in patients at the 1st day after surgery differed significantly from controls.

### 3.2. Serum miR-21 and miR-141 before and after Radical Prostatectomy

For real-time PCR applications, miR-93 exhibited the lowest coefficient of variation in the assay and was chosen as reference gene. The normalized data against miR-93 levels were reported as the mean value ± SD. 

**Figure 6:**
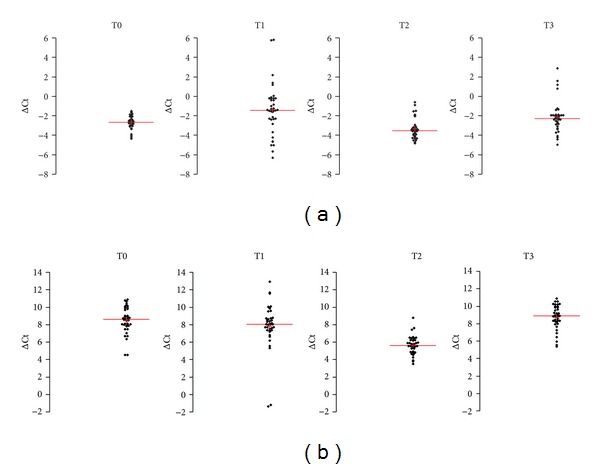
Normalized Ct (ΔCt) of miR-21 (Panel a) and miR-141 (Panel b) in serum from patients with localized prostate cancer. T0: preoperative time; T1: 1st postoperative day; T2: 5th postoperative days; T3: 30th postoperative day.

Serum levels of miR-21 and miR-141 were analyzed at T0, T1, T2, and T3 (Table 5 and Figure 6). Both miR-21 and miR-141 increased significantly at the fifth postoperative day (T0 < T2, *P* < 0.01 and *P* < 0.001, resp.). At T3, serum miR-21 and miR-141 were not significantly different from preoperative levels (T0 = T3, *P* > 0.05). 

#### 3.2.1. Comparison of Serum miR-21 and miR-141 between Patients (T0) and Healthy Controls (C)

**Figure 7:**
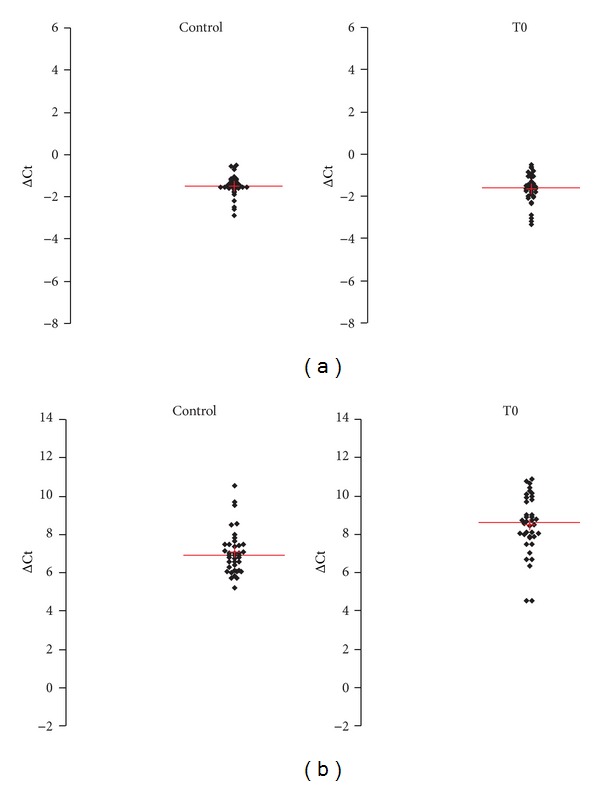
Normalized Ct (ΔCt) of miR-21 (Panel a) and miR-141 (Panel b) in sera from patients with localized prostate cancer with respect to controls. T0: preoperative time; C: control.

In prostate cancer patients serum levels of miR-21 preoperatively were not significantly different from healthy controls (*P* > 0.05). On the contrary, serum miR-141 preoperative levels were significantly lower (*P* < 0.05) than healthy controls (Figure 7 and Table 6).

Circulating levels of miR-21 and miR-141 from controls were also compared with prostate cancer group at postoperative sampling times (T1, T2, and T3). Results are shown in Table 7. For miR-21, statistically significant differences were obtained when C was compared with T2 (*P* < 0.01). Comparisons of C with T1 and T2 did not reach significance (C versus T1 and C versus T2, *P* > 0.05). Serum levels of miR-141 in control group were significantly different from those of patients only at the 30th day after surgery (C versus T3, *P* < 0.05; C versus T1 and C versus T2, *P* > 0.05).

### 3.3. Evaluation of Diagnostic Accuracy of miR-21, miR-141, hK11, and hK13

**Figure 8:**
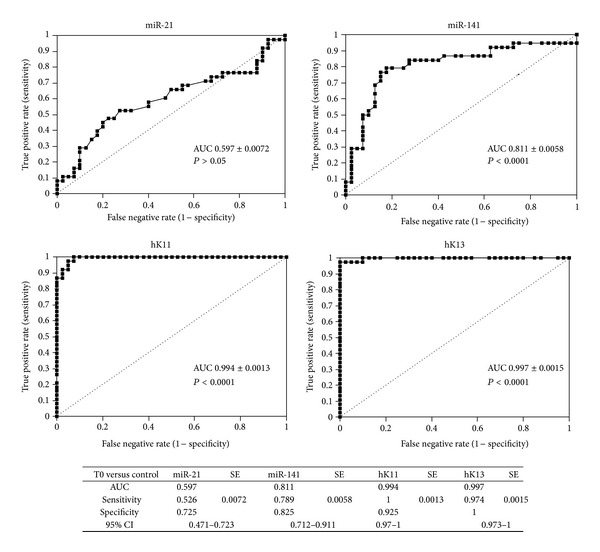
ROC curves and AUCs of serums miR-21, miR-141, hK11, and hK13 between patients (T0) and healthy controls.

The diagnostic accuracy of miR-21, miR-141, hK11, and hK13 was evaluated using Receiver Operating Characteristic (ROC) curve analysis. As shown in Figure 8, serum miR-21 displayed the lowest ability (AUC = 0.597, *P* > 0.05) to differentiate between prostate cancer patients and healthy controls, whereas miR-141 reached an AUC of 0.811 (*P* < 0.0001). Serum hK13 showed the highest diagnostic performance (AUC = 0.997, *P* < 0.0001), followed by hK11 (AUC = 0.994, *P* < 0.0001).

Logistic regression analysis was performed to evaluate the probability that the combination of miR-141 and miR-21 could discriminate prostate cancer patients from healthy controls in a better way than miR-141 alone. Area under the curve was 0.811 (which was equal to miR-141 alone) and sensitivity was lower than miR-141 (68.42% versus 78.9%), while specificity increased slightly (85% versus 82.5%).

## 4. Discussion

### 4.1. Prostate Cancer Beyond PSA: Postoperative Serum Levels of hK11 and hK13

The main diagnostic tools to detect prostate cancer include digital rectal examination, serum PSA, and transrectal ultrasound guided biopsy. PSA is a quite accurate diagnostic test, but its specificity is too low because PSA is a prostate-specific but not a prostate cancer-specific marker: for this reason, those biopsies requested for altered PSA levels are often negative. When PSA is ranged between 4.0 and 9.9 ng/mL, the first prostate biopsy is positive only up to 37% [10]. PSA belongs to the genetic family of kallikreins, a group of serine proteases widely expressed in various tissues and involved both in many physiological and in pathological processes: the alteration in their expression is related to the onset of various human diseases [11–13]. Since they are circulating proteins, kallikreins are detectable in human body fluids, such as serum, and may be used as molecular assays characterized by a low invasiveness and high accuracy. hK2 has been quite recently assayed for its utility in preoperative staging of localized prostate cancer [14]. hK2 was showed to improve the power of total, free, and intact PSA in predicting biopsy outcome in men with PSA levels [15]. Quite recently, the involvement of other kallikreins apart from PSA/hK3 and hK2 in prostate cancerogenesis has been progressively clarified: KLK11 was recently proposed as a new prognostic marker for PCa [16]. The KLK13 protein shares 51% amino acid identity with KLK11, and it is primarily expressed in mammary gland, prostate, salivary gland, and testis. KLK13 gene was found to be regulated by steroid hormones in a human breast cancer cell line so that its expression is an independent favorable prognostic marker for breast carcinoma [17].

PSA is the unique kallikrein whose postsurgical kinetics has been studied: to our knowledge, this is the first report describing postoperative changes in serum levels of other kallikreins at multiple times. Both hK11 and hK13 were significantly higher in prostate cancer patients before surgery and they significantly decreased after surgery. Serum hK13 showed a transient increase at the first day after surgery, suggesting an implication in inflammatory events, whereas serum hK11 immediately decreased after surgery. Both hK11 and hK13 stabilized at lower levels with respect to preoperative values, although they did not fall down to zero such as PSA, which became undetectable 30 days after surgery. hK11 even seemed to increase at the 30th day after radical prostatectomy, although comparisons of T3 with T2 or T1 were not statistically significant. These findings highlighted the direct correlation of these kallikreins to prostate cancer. This was confirmed by the significant difference of hK11 and hK13 serum levels in prostate cancer patients with respect to controls (T0 > C, *P* < 0.05). Serum hK11 and hK13 fluctuations after surgery appeared to have a strong correlation with prostate cancer, since their decrease to lower levels with respect to preoperative values resembles PSA postoperative decay curve.

### 4.2. Changes in Serums miR-21 and miR-141 after Radical Prostatectomy: Only a Matter of Inflammation?

microRNA are progressively emerging as crucial regulators of gene expression, since they affect the tumorigenesis as oncogenes or oncosuppressors [6, 18–20]. The intrinsic stability of miRNAs in human plasma/serum renders these molecules ideal candidates for the development of low invasive diagnostic assays [21]. The involvement of miRNAs in carcinogenesis has been reported elsewhere: this widespread interest on miRNAs led to the development of commercial cancer biomarker assays for pancreatic cancer versus pancreatitis [22], squamous versus nonsquamous lung cancer [23], and malignant pleural mesothelioma versus lung and pleura cancers [24]. miRNAs proved also to be effective in the identification of the unknown primitive cancer of metastasis [25]. Besides being involved in carcinogenesis, several studies have defined mammalian microRNAs as key regulators of the immune system. In fact, inflammation is able to regulate miRNA biogenesis [26]. 

miR-21 has been recognized as *onco*mir, and it has been demonstrated to target, among others, the following genes: tumor suppressor gene tropomyosin 1 (TPM1), programmed cell death 4 (PDC D4), maspin, phosphatase and tensin homolog (PTEN), and reversion-inducing cysteine-rich protein with kazal motifs (RECK) [26–29]. A recent study aimed to assess the expression of circulating miR-21 in serum samples from patients with different types of cancer, but without including prostate cancer because of too few cases [30]. By use of real-time quantitative reverse transcription-PCR, a marked overexpression of circulating miR-21 in 174 patients with solid cancers (breast, esophageal, gastric, colorectal, and lung cancers) with respect to 39 normal control subjects was assessed. Furthermore, miR-21 proved to be associated with the expression of genes that regulated inflammation [31]. miRNAs role in the development, maturation, and function of cells involved in the innate and adaptive immunity has been proved [32, 33]. Chen et al. analyzed the frequent deregulation of serum miRNAs in patients with lung or colorectal carcinoma: they found a quite large portion (38.5%) of the same shared members to be overexpressed also in serum samples from patients with type II diabetes. This is probably attributable to the inflammatory reactions occurring in all the samples under investigation [34]. Thus, although a set of miRNAs specifically associated with inflammation has been characterized [33], the involvement of other members may be hypothesized and still unreported.

miR-141 is representative of epithelial tissue; thus, its expression is not restricted to prostatic epithelium: nonetheless, circulating miR-141 levels in prostate cancer patients were successfully used to screen for metastatic prostate cancer with high sensitivity [6]. Virtually all epithelial cancers overexpress miR-141 (data from literature are mainly available for breast, lung, colon, and prostate cancers) [35]. The convergent deregulation of miR-141 in epithelial cancers suggests that the diagnostic performance of miR-141 in prostate cancer remains to be assessed. Furthermore, miR-141 has been shown to target p38*α*, thus modulating the oxidative stress response and ultimately affecting tumorigenesis [36]. 

In the present study, miR-21 and miR-141 levels did not show significant difference with preoperative values (*P* > 0.05) in the first postoperative day. In contrast, a significant increase of both miR-21 and miR-141 was detected at the 5th postoperative day, with respect to the preoperative time. This finding is probably attributable to systemic inflammatory reactions occurring after surgery. We hypothesize that these alterations do not appear immediately at T1 because of a *de novo* production of miRNA from other body districts. At T3, the serum levels of miR-21 and miR-141 decreased to values comparable to preoperative time (*P* > 0.05). 

Our findings on serum miR-141 seem to be in contrast with previous data in the literature. So far, miR-141 has been mainly associated with prostate cancer progression [6, 37–39]. The expression levels of circulating miRNAs in low-risk, high-risk, and metastatic castration resistant prostate cancer (mCRPC) have highlighted the characteristic overexpression of miR-141 in mCRPC compared with low-risk disease [40]. On the contrary, our analysis has been conducted on serum samples from patients with localized prostate cancer. Moreover, other discrepancies about circulating levels of miR-141 in prostate cancer have been reported by other researchers. As an example, Agaoglu and colleagues [41] did not find significant differences in circulating levels of miR-141 in prostate cancer (both localized and advanced diseases) with respect to healthy controls. Furthermore, Mahn and coworkers [42] were unable to detect serum miR-141 in localized prostate cancer: the authors hypothesized that this event is due to their patient selection criteria (they did not include patients with metastatic prostate cancer) and to the the lack of a preamplification step to enrich for miRNAs, which was instead performed by Mitchell [6]. 

Similarly to miR-141, the overexpression of miR-21 has been mainly examined in advanced cancers [43, 44]. A recent study aimed at identifying circulating miRNAs able to discriminate mCRPC from localized prostate cancer revealed a statistically significant upregulation of miR-21 in the former group [45]. On the other hand, Folini [46] found similar expressions of miR-21 in localized tumor and surrounding healthy tissue specimens from 36 patients who undergone radical prostatectomy. In summary, while the upregulation of miR-21 and miR-141 has been established in metastatic prostate cancer, their modulation in localized forms is still controversial. To our knowledge, our report is the first to make serial postoperative measurements of serum miRNAs and analysis of their postoperative kinetics. Single postoperative measurements were successfully performed by other authors, both in prostate cancer patients [42] and in other malignancies [47]. Our results were quite surprising, since both serum miR-21 and miR-141 increased significantly at 5th postoperative day to finally return to the preoperative levels. The observed fluctuations could be explained by a de novo synthesis of miRNAs, suggesting their involvement in postsurgical inflammation.

## 5. Conclusions

Our study provided innovative information about postoperative changes in the levels of circulating microRNAs and kallikreins. This was possible through serial samplings up to 30 days after surgery. 

Our results assessed that the interest regarding kallikreins should not be restricted to PSA, since both hK11 and hK13 showed a marked correlation with prostate disease. Indeed, serums hK11 and hK13 showed a statistically significant decrease after surgery, although they did not fall down to zero such as PSA. The delayed increase in expression of serums miR-21 and -141 at the 5th postoperative day could be due to a *de novo* miRNA synthesis induced by systemic inflammatory reactions. At the 30th postoperative day, serums miR-21 and miR-141 were similar to preoperative values. Overall, the results here obtained confirmed that every change in expression of serum markers after surgery should always be taken with caution and confirmed by multiple observations. The diagnostic performance of the markers under examination needs to be confirmed by larger scale studies.

## Figures and Tables

**Table 1:** Demographic characteristics of the patients enrolled in the present study.

Clinical parameters	PCa
Median age (range)	62 (54–63 ys)
Median PSA (range)	5.55 (3.11–9 ng/mL)
Clinical stage	
T1c	21 (55.3%)
T2a	6 (15.8%)
T2b	3 (7.9%)
T2c	8 (21%)
Pathological Gleason score	
6 (3 + 3)	20 (52.6%)
7 (3 + 4)	2 (5.3%)
7 (4 + 3)	12 (31.6%)
8 (4 + 4)	4 (10.5%)
Lymph node involvement	6 (15.8%)
Pathological stage	
T2a	6 (15.8%)
T2b	2 (5.3%)
T2c	24 (63.1%)
T3a	4 (10.5%)
T3b	2 (5.3%)

**Table 2:** ANOVA showing significance values of hK11 and hK13 for each comparison.

Tukey's multiple comparison test	Groups	Mean difference	*q*	*P* value	95% CI	
					Lower	Upper
hK11	T0 versus T1	162.1	7.51	*P* < 0.05	76.28	248.0
	T0 versus T2	174.7	8.09	*P* < 0.05	88.86	260.6
	T0 versus T3	115.5	5.35	*P* < 0.05	29.65	201.4
	T1 versus T2	12.58	0.59	*P* > 0.05	−73.27	98.43
	T1 versus T3	−46.63	2.16	*P* > 0.05	−132.5	39.22
	T2 versus T3	−59.21	2.74	*P* > 0.05	−145.1	26.64

hK13	T0 versus T1	−111.0	9.00	*P* < 0.05	−160.0	−61.99
	T0 versus T2	81.60	6.62	*P* < 0.05	32.59	130.6
	T0 versus T3	62.70	5.08	*P* < 0.05	13.69	111.7
	T1 versus T2	192.6	15.62	*P* < 0.05	143.6	241.6
	T1 versus T3	173.7	14.09	*P* < 0.05	124.7	222.7
	T2 versus T3	−18.90	1.53	*P* > 0.05	−67.91	30.11

T0: preoperative time; T1: 1st postoperative day; T2: 5th postoperative day; T3: 30th postoperative day.

**Table 3:** Statistical analysis of serums hK11 and hK13.

Tukey's multiple comparison test	Groups	Mean difference	*q*	*P* value	95% CI	
					Lower	Upper
hK11	C versus T0	−176.8	6.532	*P* < 0.05	−284.5	−69.23
hK13	C versus T0	−101.2	6.547	*P* < 0.05	−162.6	−39.76

T0: preoperative time; C: control.

**Table 4:** ANOVA showing significance values of hK11 and hK13 for each comparison.

Tukey's multiple comparison test	Groups	Mean difference	*q*	*P* value	95% CI	
					Lower	Upper
hK11	C versus T1	−14.72	0.544	*P* > 0.05	−122.3	−92.90
	C versus T2	−2.136	0.079	*P* > 0.05	−109.8	105.5
	C versus T3	−61.35	2.266	*P* > 0.05	−169.0	26.27

hK13	C versus T1	−212.2	13.73	*P* < 0.05	−273.6	−150.8
	C versus T2	−19.60	1.268	*P* > 0.05	−81.04	41.84
	C versus T3	−38.50	2.491	*P* > 0.05	−99.94	22.94

C: control; T1: 1st postoperative day; T2: 5th postoperative day; T3: 30th postoperative day.

**Table 5:** ANOVA showing significance values of miR-21 and miR-141 for each comparison.

Tukey's multiple comparison test	Groups	Mean difference	*q*	*P* value	95% CI	
					Lower	Upper
miR-21	T0 versus T1	−0.255	0.75	*P* > 0.05	−1.592	1.082
	T0 versus T2	1.687	4.98	*P* < 0.01	0.350	3.024
	T0 versus T3	0.498	1.47	*P* > 0.05	−0.839	1.835
	T1 versus T2	1.942	5.73	*P* < 0.01	0.605	3.279
	T1 versus T3	0.753	2.22	*P* > 0.05	−0.584	2.090
	T2 versus T3	−1.189	3.51	*P* > 0.05	−2.526	0.148

miR-141	T0 versus T1	0.666	1.75	*P* > 0.05	−0.835	2.167
	T0 versus T2	2.855	7.51	*P* < 0.001	1.354	4.356
	T0 versus T3	−0.237	0.62	*P* > 0.05	−1.738	1.264
	T1 versus T2	2.189	5.76	*P* < 0.01	0.688	3.690
	T1 versus T3	−0.903	2.37	*P* > 0.05	−2.404	0.598
	T2 versus T3	−3.092	8.13	*P* < 0.001	−4.593	−1.591

T0: preoperative time; T1: 1st postoperative day; T2: 5th postoperative day; T3: 30th postoperative day.

**Table 6:** Statistical analysis showing significance values of miR-21 and miR-141.

Tukey's multiple comparison test	Groups	Mean difference	*q*	*P* value	95% CI	
					Lower	Upper
miR-21	C versus T0	0.197	0.59	*P* > 0.05	−1.123	1.517
miR-141	C versus T0	−1.506	4.01	*P* < 0.05	−2.988	−0.024

T0: preoperative time; C: control.

**Table 7:** ANOVA showing significance values of miR-21 and miR-141 for each comparison.

Tukey's multiple comparison test	Groups	Mean difference	*q*	*P* value	95% CI	
					Lower	Upper
miR-21	C versus T1	−0.058	0.17	*P* > 0.05	−1.378	1.262
	C versus T2	1.884	5.63	*P* < 0.01	0.564	3.204
	C versus T3	0.695	2.08	*P* > 0.05	−0.625	2.015

miR-141	C versus T1	−0.840	2.24	*P* > 0.05	−2.322	0.642
	C versus T2	1.349	3.59	*P* > 0.05	−0.133	2.831
	C versus T3	−1.743	4.64	*P* < 0.05	−3.225	−0.261

C: control; T1: 1st postoperative day; T2: 5th postoperative day; T3: 30th postoperative day.
